# Cyclosporine Effects on Pediatric Kidney Recipients

**DOI:** 10.5812/numonthly.2659

**Published:** 2012-03-01

**Authors:** Maryam Hami

**Affiliations:** 1 Department of Internal Medicine, Mashhad University of Medical Sciences, Mashhad, IR Iran

**Keywords:** Cyclosporine, Pediatrics, Kidney

Dear Editor,

I read with interest the article by Beiraghdar et al. (1), entitled “Cyclosporine trough and 2 hour post dose monitoring and its contributing factors among pediatric kidney recipients,” recently published in the Nephro-Urology Monthly. The authors correctly mentioned that there is a negative relationship between cyclosporine A levels (C0 and C2) and serum creatinine. Based on their data, the mean of C0 levels (118 ± 65 ng/mL) was in the therapeutic range, which is an acceptable level of cyclosporine after 6 months using the EMIT (Enzyme multiplied immunoassay technique) assay method (2). Then lower serum creatinine levels that better reflects graft function are predictable. Although in this study, the mean of C2 levels (471 ± 148 ng/mL) was lower than therapeutic levels (600-800 ng/mL), patients had good graft function. This result can be explained by previous similar studies in Iran in which most patients at therapeutic C2 levels presented with cyclosporine nephrotoxicity or the recipients did not reach target levels, so they didn’t recommend routine checking of C2 levels (2, 3). This may be due to genetic differences in drug metabolism in different races which can lead to different peak levels or different peak times (for example 3 or 4 hours after taking) in Iranian patients.

Another factor that may impact on graft function consists of the donor’s demographic data such as type (cadaveric vs. living), age, and/or gender. Based on previous studies, the long-term graft survival rate is significantly higher from living-related or unrelated donors than in the cadaveric group (4). Krajewska et al. reported that the best graft functions were found in women who had received male donors’ kidneys and they also showed that older donors impaired remote graft outcomes irrespective of cyclosporine blood levels (5). In my opinion the effects of the donors’ age on C2 levels in this article needs more discussion and further research.

Episodes of hepatic dysfunction that is revealed by serum aminotransferase elevation levels may occur at higher doses of cyclosporine (1). As this article only reported a significant relationship between C0 level and serum Alanine Aminotransferase, then maybe we can conclude that C0 level is a better marker of drug dosage at least in Iranian patients.

In a previous article in Iran, Hami et al. said that there was no significant relationship between C0 levels and blood glucose or blood pressure (6). The authors of this article reported similar results. They did not find any significant correlation between hemoglobin, uric acid, fasting blood glucose and serum cholesterol with C0 and C2 levels.

Finally, I would like to suggest that C0 levels may be a more practical and accurate marker of kidney function and dose adjustment in Iranian patients until the true peak level can be determined.
